# Gut epithelial barriers as regulators of metabolite-driven immune responses

**DOI:** 10.3389/fimmu.2026.1959305

**Published:** 2026-10-27

**Authors:** Anuradha Tyagi, Rajul Tayal, Dharmendra Nath Bhatt, Shivani Srivastava, Anil Kumar, Antresh Kumar, Vinay Kumar

**Affiliations:** 1 College of Medicine, Pennsylvania State University Hershey Medical Centre, Hershey, PA, United States; 2 National Institute of Plant Genome Research, New Delhi, India; 3 Molecular Pathology Section, Laboratory of Immunogenetics, Division of Intramural Research/National Institute of Allergy and Infectious Diseases (NIAID)/National Institutes of Health (NIH), Bethesda, MD, United States; 4 Department of Cellular and Molecular Physiology, Yale University School of Medicine, New Haven, CT, United States; 5 University Centre for Research and Development, Chandigarh University, Mohali, Punjab, India; 6 Department of Biochemistry, Central University of Haryana, Mahendergarh, India

**Keywords:** epithelial barrier, immune function, inflammation, microbial metabolites, microbiota

## Abstract

Immunometabolism provides a framework for understanding how metabolic cues shape immune cell fate and function. While traditionally centered on cell-intrinsic pathways, emerging evidence highlights a critical role for epithelial barrier regulation in shaping immune responses by controlling exposure to microbiome derived metabolites. Microbial metabolites including short-chain fatty acids, bile acid derivatives, tryptophan catabolites, and polyamines engage conserved metabolic checkpoints such as mTOR, AMPK, PPARs, SIRT1, and HIF-1α to influence immune activation, tolerance, and inflammatory tone. Critically, epithelial barriers regulate immune exposure to these signals, positioning barrier integrity as an upstream determinant of immunometabolic reprogramming. Altered epithelial metabolism compromises tight junction stability and metabolite flux, contributing to dysregulated immune activation and chronic inflammation. In this review, we synthesize current evidence supporting the concept that epithelial barriers act as metabolic gatekeepers of immunity, integrating microbial signals with host metabolic pathways to regulate immune function and disease susceptibility.

## Graphical Abstract

**Figure:**
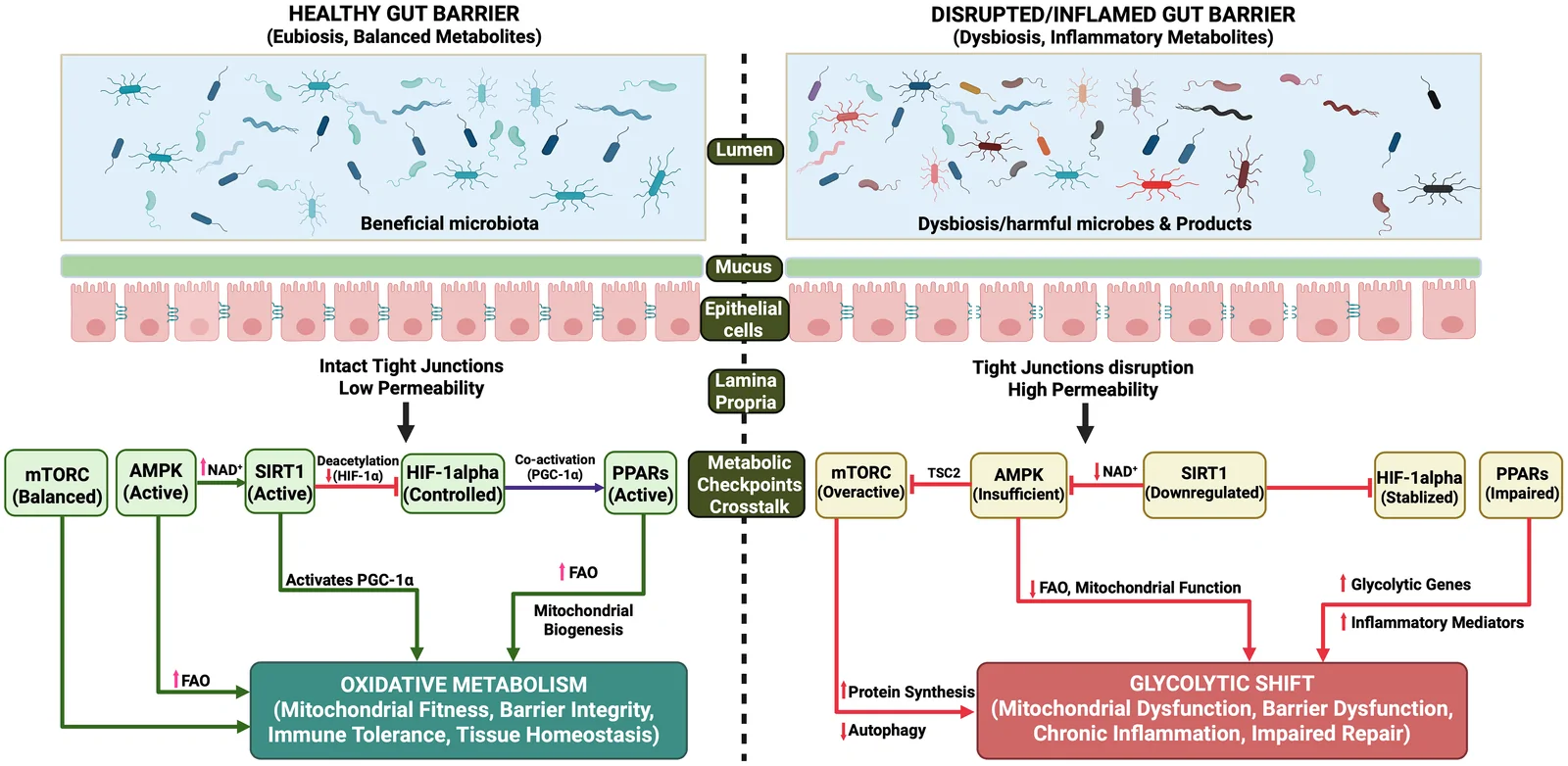
Created in BioRender. Kumar, V. (2026) https://BioRender.com/noaodx5.

## Highlights

- Epithelial barriers act as metabolic gatekeepers that regulate immune exposure to microbial and dietary metabolites.
- Microbiome-derived metabolites influence immune cell function through conserved metabolic checkpoints such as mTOR, AMPK, PPARs, SIRT1, and HIF-1α.
- Disruption of epithelial metabolic balance alters tight junction stability and drives maladaptive immunometabolic responses.

## 1 Introduction

Immunometabolism has arisen as a comprehensive framework for comprehending how metabolic pathways influence immune cell destiny, functionality, and inflammatory capacity. Cellular metabolic programs function not just as bioenergetic support but also as regulatory systems that integrate nutrition availability, environmental stress, and signaling cues to govern immune activation, differentiation, and effector responses. Metabolic processes are evolutionarily more ancient than specialized immune functions, and many components of immune regulation are thought to have emerged through the adaptation and co-option of conserved metabolic pathways (1). Importantly, immune cell metabolic states are highly dynamic and metabolically plastic, with glycolytic and oxidative pathways functioning cooperatively and flexibly according to activation state, tissue environment, nutrient availability, and inflammatory signals.

A hallmark of immunological activation is metabolic transformation. The activation of antigen receptors and innate pattern recognition receptors triggers coordinated signaling via pathways including PI3K-AKT-mTOR, NF-κB, and MAPK, leading to a swift reconfiguration of nutrition usage and biosynthetic processes (2, 3). These modifications allow immune cells to fulfill the energetic and anabolic requirements of proliferation, cytokine synthesis, and cytotoxic activity. Rather than representing rigid metabolic states, glycolytic and oxidative programs are dynamically balanced and frequently coexist during immune activation and resolution. Metabolites produced in these pathways, including glucose-derived intermediates, fatty acids, and tricarboxylic acid cycle components like succinate, serve as signaling molecules that modulate transcriptional and epigenetic programs, thereby refining immune responses in a context-dependent manner (4).

Dysregulation of immunometabolic pathways contributes to a wide range of chronic inflammatory and immune mediated disorders, including obesity related metaflammation, autoimmunity, cancer, and chronic infection (5–7). In these varied states, immunological failure often indicates maladaptive activation of conserved metabolic checkpoints instead of disease-specific pathways, underscoring immunometabolism as a fundamental organizing element of pathogenesis.

The gut microbiota has emerged as a major source of such signals. As a metabolically active system, it produces a diverse array of bioactive metabolites, including short-chain fatty acids (SCFAs), bile acid derivatives, polyamines, and tryptophan catabolites, which interact with host receptors to modulate immune cell metabolism and function (8, 9). Importantly, the immunological impact of these metabolites is determined not only by their production but also by their access to host tissues. The intestinal epithelial barrier plays a central role in this process. By regulating paracellular permeability through tight junctions, the epithelium controls the spatial and temporal exposure of immune cells to luminal metabolites and microbial products (10). Barrier integrity serves as a metabolic gatekeeper that connects microbial activity to immune metabolic programming. Disruption of epithelial metabolic balance alters tight junction stability and metabolite flux, leading to inappropriate immune activation and chronic inflammation. In this review, we focus on epithelial barriers as key regulators of immunometabolism. We synthesize current evidence demonstrating how epithelial metabolic state governs barrier integrity, controls metabolite exposure, and shapes downstream immune responses. We propose that barrier regulated access to microbial metabolites represents a central mechanism linking metabolism to immune regulation and disease.

## 2 Metabolic checkpoints governing immune cell fate

Activation, differentiation, and persistence of immune cells are regulated by conserved metabolic checkpoints that integrate nutrient availability, environmental signals, and cellular stress, thereby influencing immune outcomes. In addition to bioenergetic regulation, these checkpoints serve as signaling centers that link metabolism to transcriptional and epigenetic processes within adaptive, innate, and barrier associated immune compartments (11–13). Among these, Mammalian target of rapamycin (mTOR), AMP-activated protein kinase (AMPK), Hypoxia-inducible factor-1α (HIF-1α), Peroxisome proliferator-activated receptors (PPARs), and the NAD^+^ dependent deacetylase Sirtuin 1 (SIRT1) have emerged as central regulators of immunometabolic decision making.

mTOR signaling functions as a crucial sensor of nutrient availability and growth signals, facilitating anabolic metabolism, glycolysis, and the biosynthetic pathways essential for effector immune responses (14) whereas AMPK activation during energetic stress promotes catabolic metabolism, mitochondrial function, and regulatory immune states (15, 16). Rather than functioning as strictly opposing metabolic states, these pathways operate within flexible and adaptive metabolic networks that allow immune cells to dynamically adjust inflammatory, regulatory, and tissue repair responses according to environmental and nutritional conditions.

Similarly, HIF-1α synchronizes oxygen availability with glucose metabolism and inflammatory signals to influence immune cell activity during metabolic stress (17). The stabilization of HIF-1α in hypoxic or inflamed tissues facilitates glycolytic reprogramming and inflammatory effector functions, establishing it as a pivotal coordinator of metabolic adaptability in immunity (17, 18). In addition to immune cells, HIF-1α facilitates epithelial adaptation to hypoxic and inflammatory stress, hence maintaining barrier integrity and tissue tolerance via hypoxia-responsive metabolic pathways (19). Nuclear receptors, such as PPARγ and metabolic regulators including SIRT1 further modulate immune metabolism by promoting oxidative metabolism, anti-inflammatory programs, and stress adaptation (20–22). PPARγ supports fatty acid oxidation and anti-inflammatory immune programs, contributing to M2-like macrophage polarization, immune regulation, and tissue repair (23, 24). SIRT1 links cellular energy status to transcriptional and epigenetic regulation through NAD^+^-dependent deacetylation, thereby restraining inflammatory signaling and supporting mitochondrial function and regulatory immune responses (20–22).

In T cells, activation is accompanied by metabolic reprogramming that supports proliferation, differentiation, and effector function, whereas regulatory T-cells (Tregs) preferentially engage mitochondrial metabolism and fatty acid oxidation to maintain suppressive activity and immune tolerance (25, 26). Importantly, these metabolic programs are not fixed, and T-cell subsets exhibit considerable metabolic plasticity in response to tissue environment, nutrient availability, and inflammatory signals (25). Similarly, macrophage polarization is associated with distinct but overlapping metabolic adaptations, with pro-inflammatory M1-like macrophages characterized by enhanced glycolytic and HIF-1α associated pathways, whereas anti-inflammatory M2-like macrophages preferentially utilize oxidative and lipid associated metabolism regulated in part by PPAR signaling (27). Dendritic cells likewise undergo metabolic reprogramming that influences their immunological function, as glycolytic activation supports inflammatory antigen-presenting states, whereas oxidative metabolism and mitochondrial fitness contribute to tolerogenic phenotypes and immune regulation (28, 29). Together, these pathways integrate metabolic information into immune cell functional programs.

Importantly, these conserved metabolic checkpoints are not restricted to immune cells but are also active within intestinal epithelial cells (IECs), where they integrate nutrient availability, microbial metabolites, oxygen tension, and cellular stress to coordinate barrier function and immune communication. Through these pathways, IECs regulate the quantity and composition of microbial metabolites, antigens, and inflammatory ligands that reach the lamina propria. Consequently, epithelial metabolic status directly influences neighboring immune populations, including macrophages, dendritic cells, and lymphocytes. Metabolically competent IECs maintain barrier function, limit microbial translocation, and support tolerogenic macrophage polarization and regulatory immune responses (30). In contrast, epithelial metabolic dysfunction promotes permeability, enhanced exposure of lamina propria immune cells to microbial products, and increased production of inflammatory mediators, thereby amplifying inflammatory macrophage activation and downstream immune responses (31, 32). These observations suggest that metabolic checkpoints operate across both epithelial and immune compartments, linking epithelial metabolic state to immune cell programming.

Although these checkpoints govern how immune cells respond to metabolic inputs, their activation ultimately depends on the availability and accessibility of metabolites. Microbiome derived metabolites and nutrient signals engage these pathways only when they gain access to immune compartments. Thus, the immunological impact of metabolic checkpoints is not solely determined by intracellular signaling but also by upstream mechanisms that regulate metabolite exposure. In this way, epithelial barriers emerge as key regulators of immunometabolic signaling by controlling the spatial and temporal access of metabolites and microbial products to immune cells. This barrier-controlled metabolite exposure provides the link between microbial metabolism and immune cell programming, positioning metabolic checkpoints as downstream effectors of barrier regulated immune responses. The integration of metabolic programs, metabolite derived signals, and conserved metabolic checkpoints that collectively shape immune cell fate is summarized in **Figure 1**.

**Figure 1:**
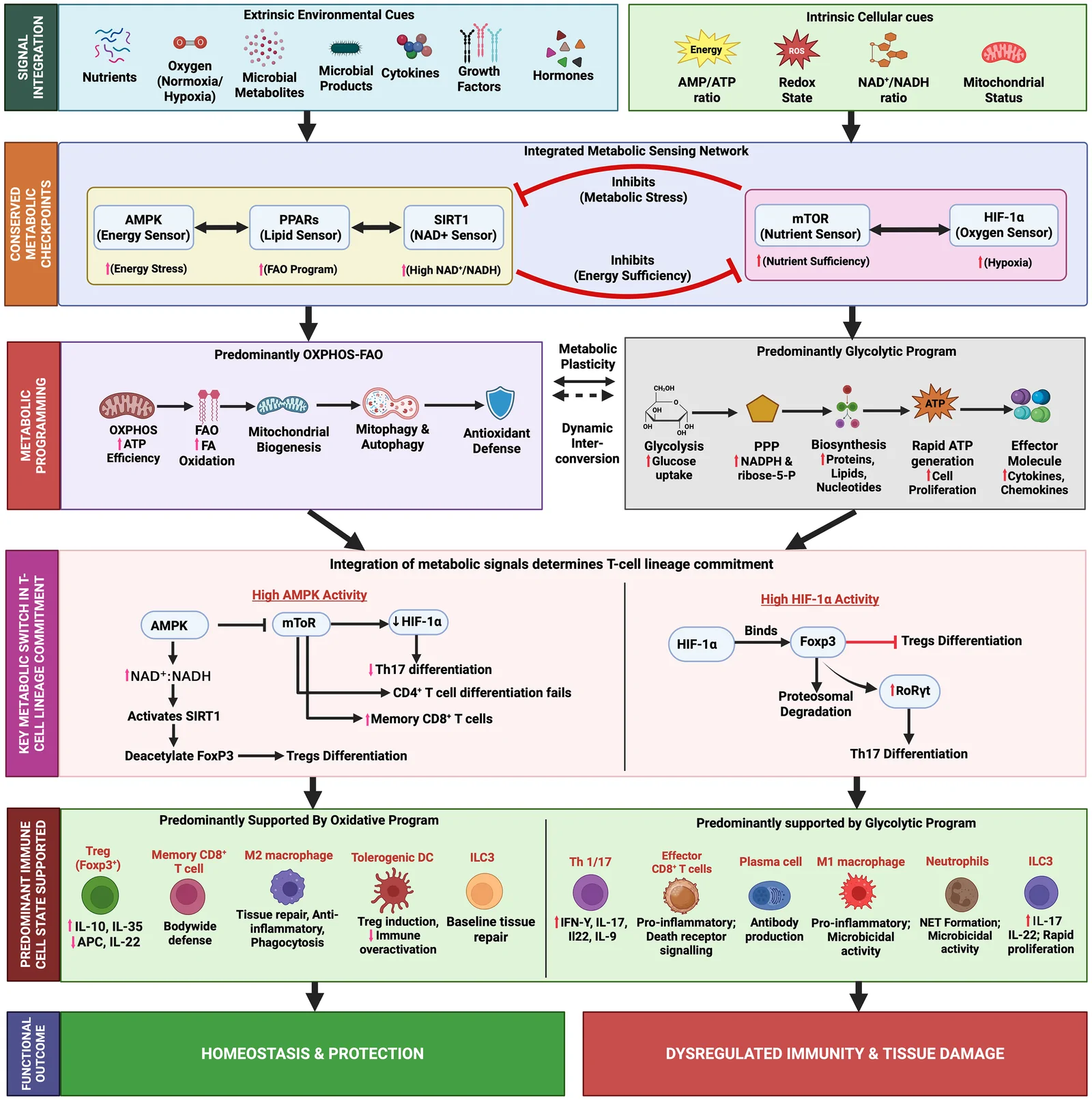
Conserved metabolic checkpoints regulate immune cell metabolic programming and functional specialization. Extrinsic environmental cues, including nutrients, oxygen availability, microbiota derived metabolites, microbial products, cytokines, growth factors, and hormones, together with intrinsic cellular cues, including AMP/ATP ratio, redox state, NAD^+^/NADH ratio, and mitochondrial status, are integrated by the conserved metabolic checkpoints AMPK, PPARs, SIRT1, mTOR, and HIF-1α. Coordinated checkpoint activity establishes predominantly oxidative or predominantly glycolytic metabolic programs. Activation of the AMPK-PPAR-SIRT1 axis promotes OXPHOS, FAO, mitochondrial biogenesis, mitophagy, and antioxidant defense, whereas activation of the mTOR-HIF-1α axis enhances glycolysis, pentose phosphate pathway (PPP) activity, biosynthesis, and inflammatory effector functions. The balance between these metabolic programs regulates T-cell lineage commitment. AMPK activation suppresses mTOR-HIF-1α signaling, activates SIRT1, stabilizes Foxp3, and promotes regulatory T-cell (Treg) differentiation and memory CD8^+^ T-cell development. In contrast, HIF-1α promotes RORγt expression while inducing Foxp3 degradation, thereby favoring Th17 differentiation. Consequently, oxidative metabolism predominantly supports Tregs, memory CD8^+^ T cells, M2 macrophages, tolerogenic dendritic cells, ILC3s, and epithelial barrier maintenance, whereas glycolytic metabolism predominantly supports Th1, Th17, and Th9 cells, effector CD8^+^ T cells, plasma cells, M1 macrophages, activated dendritic cells, and neutrophils. Together, these metabolic programs determine whether immune responses support tissue integrity and repair or drive inflammatory pathology. Created in BioRender. Kumar, V. (2026) https://BioRender.com/noaodx5.

## 3 Microbiome derived metabolites as regulators of immune function

The gut microbiome acts as a dynamic metabolic organ, continuously producing bioactive metabolites that convey environmental, dietary, and microbial signals to the host immune system. These compounds converge on conserved metabolic checkpoints including AMPK, mTOR, HIF‑1α, PPARs, and SIRT1 modulating immune cell differentiation, effector functions, and tissue specific responses (33, 34). Key microbial metabolites, their dominant microbial sources, immunometabolic effects, and disease associations are summarized in **Table 1**.

**Table 1:** Major microbiome derived metabolites and their immune effects.

Category	Molecule/product	Primary source	Immunometabolic effect	Disease relevance	References
Microbiome-derived metabolites	Butyrate (SCFA)	Firmicutes (*Faecalibacterium*, *Roseburia*)	Promotes Treg differentiation; inhibits NF-κB; enhances OXPHOS; HDAC inhibition	IBD, obesity, insulin resistance	(35, 36)
	Propionate (SCFA)	Bacteroidetes	Regulates appetite and gluconeogenesis; suppresses inflammatory cytokine production	Obesity, metabolic syndrome	(37)
	Acetate (SCFA)	*Bacteroidetes*, *Prevotella* spp., *Bifidobacterium* spp., *Akkermansia muciniphila*	Systemic energy substrate; enhances memory CD8^+^ T-cell metabolism; modulates inflammatory tone	Infection, cancer, metabolic disease	(38–40)
	Secondary bile acids (deoxycholic acid, lithocholic acid)	*Clostridium* spp. (BSH^+^, 7α-dehydroxylating bacteria)	Modulate macrophage polarization; regulate Th17/Treg balance via FXR/TGR5 signaling	Autoimmunity, cancer, metabolic disease	(41, 42)
	Indole derivatives (indole-3-lactate, indole-3-propionic acid, indole-3-acetate)	*Lactobacillus*, *Bacteroides*, *Peptostreptococcus* spp.	Enhance epithelial barrier integrity; restrain Th17 differentiation; regulate innate lymphoid cells via AhR	IBD, mucosal inflammation	(43–45)
	Polyamines (cadaverine, putrescine, spermidine)	Enterobacteriaceae	Dose-dependent immune metabolic and epigenetic reprogramming; regulate redox balance and chromatin accessibility	Colitis, intestinal inflammation	(46, 47)
	Succinate	Bacteroidetes and Firmicutes	Activates HIF-1α-dependent inflammatory macrophage programs; promotes intestinal inflammation	Inflammation, cancer, intestinal disorders	(4, 48)
	Lactate	*Lactobacillus* spp.*;* anaerobic microbiota	Regulates T-cell metabolism, migration, and effector function via NAD(H)-dependent metabolic rewiring	Cancer, chronic inflammation	(49, 50)
Host-derived, microbiota-influenced metabolites	Kynurenine	Host IDO1/TDO2-dependent tryptophan metabolism influenced by inflammatory and microbiome-associated signals	Suppresses T-cell infiltration via AhR–STAT1–CXCL9/CXCL10 signaling; modulates immune trafficking and inflammatory tone	Tuberculosis, cancer, autoimmunity	(51, 52)
Microbial structural products/PAMPs	Lipopolysaccharide (LPS)	Gram-negative bacteria	Activates TLR4–NF-κB signaling; induces insulin resistance, inflammasome activation, and inflammatory cytokine production	Obesity, metabolic syndrome, NAFLD	(53–55)
	Lipoteichoic acid (TLR2 ligands)	*Lactobacillus*, *Bifidobacterium*, and *Clostridium* spp.	Activate TLR2 signaling; stimulate antimicrobial defense pathways; enhance epithelial barrier responses	IBD, mucosal inflammation, infection	(56, 57)
	Flagellin (TLR5 ligand)	Flagellated bacteria including *Salmonella*, *Escherichia coli*, and commensal motile bacteria	Activates TLR5 signaling; induces antimicrobial peptide production; promotes epithelial defense and mucosal immune surveillance	IBD, enteric infection, mucosal inflammation	(58, 59)

SCFAs such as acetate, propionate, and butyrate (contribute to 90 - 95% of intestinal SCFAs), generated from dietary fiber fermentation, exemplify this integration (60, 61). They serve as energy substrates while signaling through GPR41/43/109A and inhibiting histone deacetylases, promoting oxidative phosphorylation (OXPHOS) and fatty acid oxidation in Tregs, stabilizing FOXP3, and reinforcing immune tolerance (62, 63). In immune cells, SCFAs promote oxidative and lipid-associated metabolic programs linked to Treg stability, FOXP3 expression, and immune tolerance, while simultaneously restraining NF-κB driven inflammatory signaling in macrophages, resulting in reduced TNF-α and IL-6 production (35, 64, 65). Importantly, these effects are contingent on controlled epithelial permeability, which regulates the availability of SCFAs to immune compartments. Disruption of barrier integrity alters SCFA gradients and may shift their effects from regulatory to pro-inflammatory outcomes (65, 66).

Bile acid metabolites extend this network by linking lipid metabolism to immune regulation. Secondary bile acids engage FXR, TGR5, and other receptors to promote anti-inflammatory M2 macrophage polarization and modulate Th17/Treg differentiation via mitochondrial respiration and ROS, illustrating how diverse microbial molecules converge on shared checkpoints to coordinate host immunity (8, 41, 67). Bile acid signaling regulates immune responses through receptor and tissue specific pathways that influence macrophage polarization, T-cell differentiation, and epithelial integrity. The immunological effects of bile acids are similarly influenced by their regulated transport across epithelial barriers, which governs their local and systemic availability. Alterations in barrier function may therefore reshape bile acid mediated immune signaling and contribute to inflammatory disease.

Tryptophan metabolism generates a wide spectrum of bioactive metabolites through host and microbial pathways that modulate immune function and support epithelial integrity. Microbiota derived indoles provide an important layer of regulation by activating AhR, sustaining IL-22 production in ILC3s, enhancing epithelial barrier integrity, and restraining Th17 responses (68). Among these metabolites, microbiota derived indole-3-propionic acid (IPA) specifically stimulates mitochondrial respiration in CD4^+^ T cells, augmenting fatty acid and amino acid oxidation, fostering anti-inflammatory phenotypes, and safeguarding against intestinal inflammation (69). Notably, indole metabolites also act directly on epithelial cells to strengthen tight junctions and mucus barrier function, thereby reinforcing barrier integrity and limiting inappropriate immune exposure to luminal signals (70). Emerging evidence further indicates that epithelial cells actively regulate metabolite accessibility through specialized transport systems, including monocarboxylate transporter 1, which mediates luminal lactate uptake and influences epithelial metabolism (71), and the Ostα-Ostβ transporter complex, which controls intestinal bile acid transport and maintains bile acid balance (72). Together, these findings highlight reciprocal interactions between microbial metabolism and epithelial barrier function, whereby microbial metabolites reinforce epithelial integrity, while epithelial barrier properties and transport mechanisms regulate the distribution and accessibility of microbial metabolites to immune compartments. In contrast, inflammatory signals can redirect host tryptophan metabolism toward the kynurenine pathway, generating metabolites that suppress effector T-cell responses and promote regulatory programs (43, 73, 74). Polyamines such as spermidine and cadaverine further integrate redox, mitochondrial, and chromatin signaling, promoting adaptive or inflammatory immune responses depending on concentration, tissue environment, and metabolic state. Emerging microbial metabolites including lactate, succinate, NAD^+^ precursors, urolithins, and sphingolipids similarly modulate HIF‑1α, SIRT1, and AMPK, influencing barrier integrity, chronic inflammation, and antitumor immunity (44, 75, 76).

Collectively, microbial metabolites influence immune responses most effectively when integrated with epithelial mechanisms that regulate their distribution and access to immune compartments. This barrier-controlled metabolite exposure provides a critical link between microbial activity and immune regulation, forming the basis for the epithelial gatekeeping mechanisms discussed in subsequent sections. The multiscale integration of microbiome derived metabolites, epithelial barrier function, and systemic metabolic relays that together coordinate immune responses across tissues is depicted in **Figure 2**.

**Figure 2:**
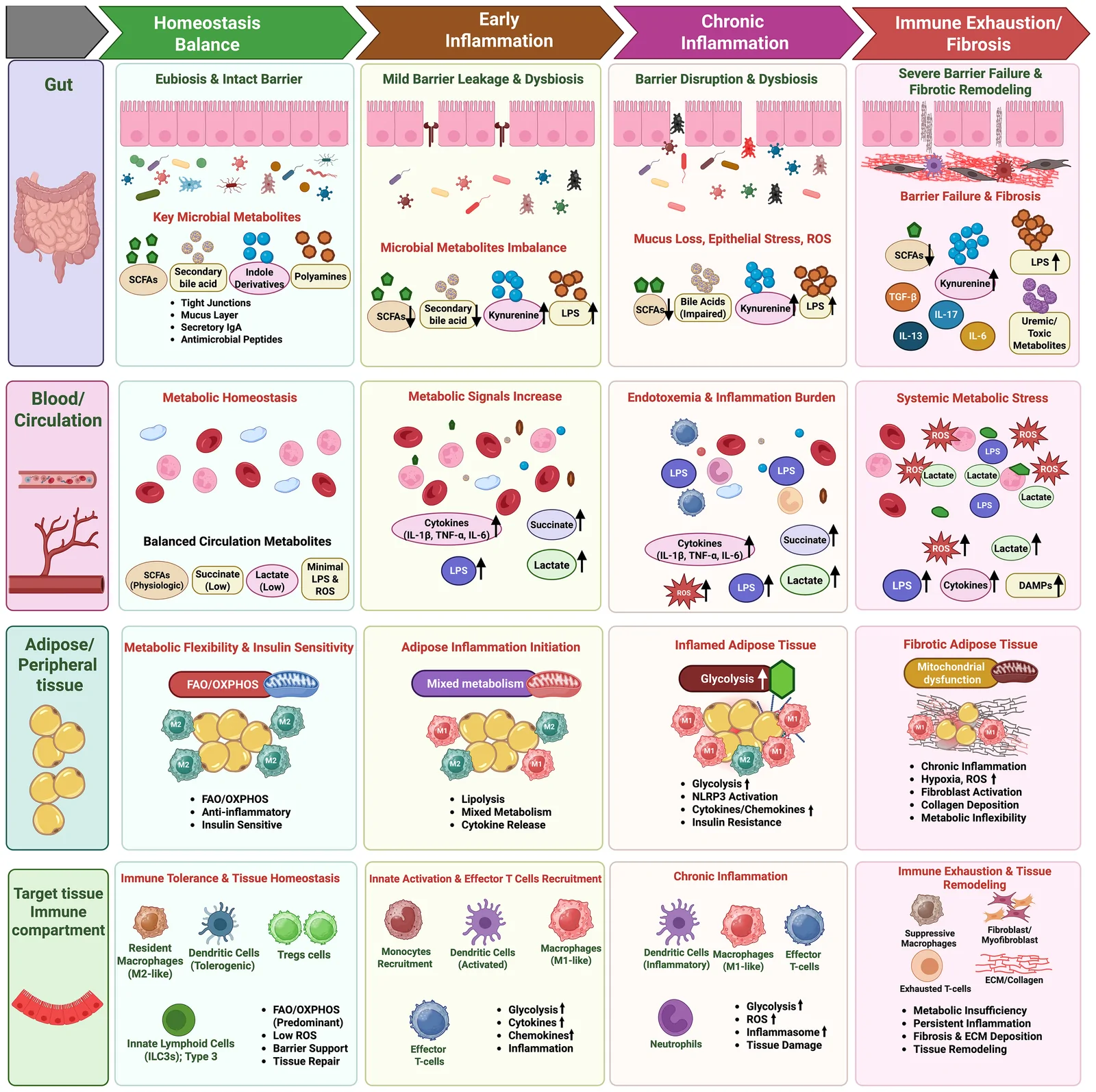
Multiscale integration of microbial and systemic metabolic signals in immune regulation. Microbial metabolite availability, epithelial barrier status, and circulating metabolic cues are integrated across spatial and temporal scales to shape immune fate. Under normal physiological conditions, regulated access to microbiome-derived metabolites facilitates balanced tissue immunometabolism. Initial barrier disruption enhances selective metabolite and platelet derived lactate/succinate signaling in the bloodstream, triggering inflammatory metabolic reprogramming. Progressive barrier disruption and endotoxemia exacerbate systemic metabolic stress, leading to glycolytic dominance, inflammasome activation, and immunological dysfunction in peripheral and inflammatory tissues. Together, this schematic illustrates how barrier-controlled microbial signals and systemic metabolic relays coordinate immune tolerance, inflammation, and exhaustion across disease progression. Created in BioRender. Kumar, V. (2026) https://BioRender.com/noaodx5.

## 4 Epithelial barriers as metabolic gatekeepers

The intestinal epithelium constitutes a metabolically active interface that determines how microbial and dietary cues are translated into immune instruction. Rather than functioning as a static physical boundary, the epithelial barrier dynamically regulates paracellular permeability and thus governs the dose, timing, and anatomical context in which microbial metabolites and microbial products gain access to immune cells in the lamina propria. This regulatory function positions the epithelium as a critical metabolic gatekeeper that shapes host-microbiota communication and influences mucosal immunity and disease susceptibility. Supporting this concept, Kaiko et al, (77) demonstrated that the compartmentalized architecture of the colonic crypt restricts the exposure of crypt-resident stem cells to microbiota derived metabolites, with differentiated colonocytes acting as a protective metabolic barrier. Mechanistically, epithelial gatekeeping is mediated through coordinated regulation of paracellular permeability, selective transporter mediated metabolite flux, and epithelial metabolic sensing pathways, which together determine the concentration, specificity, and temporal exposure of metabolites to immune compartments. Although the intestinal epithelium has long been recognized as a gatekeeper regulating the passage of luminal contents into host tissues, emerging evidence indicates that this function is actively shaped by epithelial metabolic state and stress-response signaling pathways (78).

Tight junctions are central to this gatekeeping function, yet their stability reflects active cellular regulation rather than passive structural assembly. Recent work has demonstrated that epithelial stress adaptation pathways directly control tight junction maintenance (79). PEAK1, a pseudokinase enriched at cell-cell contacts, interacts with the tight junction scaffold protein ZO-1 and shields it from autophagy mediated degradation. Loss of PEAK1 exposes a LC3-interacting region on ZO-1, promoting selective autophagic turnover of junctional components, increased epithelial permeability, and heightened susceptibility to colitis (79).

Epithelial cells sense nutrient and metabolic stress through conserved signaling pathways that integrate energy availability, oxygen tension, and redox balance. AMPK responds to cellular energy deficiency by promoting tight junction assembly and autophagy, thereby reinforcing epithelial stability under metabolic stress (80, 81). In addition, AMPK mediated activation of PINK1/Parkin dependent mitophagy preserves mitochondrial function and alleviates oxidative stress induced epithelial injury, highlighting a key link between energy sensing, mitochondrial quality control, and mucosal integrity (82). In parallel, HIF-1α enables epithelial adaptation to low oxygen conditions, supporting mucosal protection through regulation of junctional organization and stress-response pathways (83–85). Conversely, mTOR signaling reflects nutrient abundance and regulates epithelial growth and polarity, with disruption of mTOR pathways leading to epithelial defects and impaired permeability control (86, 87). Autophagy related pathways, including BECN1 dependent processes, further support epithelial integrity by regulating junctional turnover, stress responses, and mucus secretion from intestinal goblet cells (88, 89). Recent evidence further demonstrates that epithelial-intrinsic signaling pathways actively regulate mucosal integrity, as intestinal epithelial cell specific deletion of JAK2 increases intestinal permeability, alters tight junction associated gene expression, and promotes low-grade inflammatory activation, thereby highlighting the importance of epithelial signaling networks in maintaining barrier integrity and immune regulation (31). Mitochondrial function and redox balance provide an additional layer of regulation, as metabolic dysfunction impairs epithelial bioenergetic processes and promotes intestinal inflammation; notably, epithelial NAD^+^ depletion drives mitochondrial dysfunction and inflammatory signaling, linking metabolic disturbances to epithelial injury and immune activation (32). Together, these pathways enable epithelial cells to dynamically adjust permeability in response to metabolic cues, thereby regulating immune access to luminal metabolites.

Beyond epithelial-intrinsic mechanisms, microbial metabolites constitute a major regulatory layer that governs intestinal epithelial cell (IEC) function and preserves barrier integrity through host sensing pathways. SCFAs, particularly butyrate, serve not only as the primary oxidative substrate for colonocytes but also as signaling molecules that regulate epithelial gene expression through histone deacetylase inhibition and activation of G protein-coupled receptors, including FFAR2 (GPR43), FFAR3 (GPR41), and GPR109A (90–92). Through these mechanisms, SCFAs promote epithelial differentiation, stimulate mucus production, enhance tight junction assembly, and support barrier restitution following injury. In parallel, butyrate suppresses excessive NF-κB dependent inflammatory signaling while promoting AMPK activation, thereby preserving epithelial integrity and limiting inappropriate immune activation (36, 90). Microbial metabolites also regulate epithelial inflammasome pathways that coordinate barrier maintenance and host-microbiota interactions. NLRP6 inflammasome activation within IECs promotes IL-18 secretion, antimicrobial peptide production, goblet-cell mucus release, and epithelial regeneration, thereby maintaining barrier integrity and microbial ecological balance (93, 94). Disruption of these pathways alters microbial community composition, increases intestinal permeability, and enhances susceptibility to inflammatory disease (93, 94). In addition, microbiota-derived tryptophan metabolites activate AhR signaling, stimulating IL-22 dependent epithelial repair programs, antimicrobial defense, and mucosal protection (45). A crucial connection between microbial metabolism and epithelial gatekeeping is the communication via the AhR, which synchronizes luminal metabolic signals with epithelial stress-adaptation mechanisms. Indole-3-propionic acid, a microbial metabolite generated from tryptophan, stimulates AhR to increase the production of tight junction proteins such as occludin, claudin-1, and ZO-1, consequently fortifying epithelial integrity, decreasing intestinal permeability, and restricting LPS transfer (95). Beyond AhR, epithelial cells integrate microbial metabolites through additional sensing systems, including xenobiotic receptors such as PXR, bile acid receptors such as FXR and TGR5, and pattern-recognition pathways such as Toll-like receptors, which collectively regulate permeability, transporter expression, and mucosal immune signaling (96, 97). Together, these sensing mechanisms link luminal metabolic cues to transcriptional programs that coordinate epithelial structure with immune responses.

Selective transporter mediated processes further refine epithelial gatekeeping by controlling the uptake and trans-epithelial flux of microbial metabolites. Transport systems such as monocarboxylate transporters (MCT1/SLC16A1 and MCT4/SLC16A3) regulate metabolite availability to immune compartments and can themselves be modulated by metabolites such as butyrate (98). In parallel, microbial metabolites exert diverse effects on epithelial physiology through receptor-mediated pathways. For example, bile acids can either strengthen or disrupt tight junction organization depending on concentration and receptor engagement, thereby dynamically modulating permeability (99). Sulfated secondary bile acids further influence epithelial integrity and inflammatory signaling, linking metabolite sensing to immune regulation (100). In addition, microbiome derived metabolites influence epithelial cell-cell interactions, junctional organization, and immune responses to infection, highlighting their role in coordinating mucosal defense with barrier regulation (101). Together, these transport and sensing mechanisms enable epithelial cells to selectively regulate metabolite access at mucosal surfaces, reinforcing their role in controlling immune signaling.

Epithelial regulation is further shaped by reciprocal immunometabolic interactions between epithelial cells and immune populations. In people living with HIV, immunometabolic defects in CD8^+^ T cells are associated with impaired gut barrier integrity and increased microbial translocation, indicating that immune cell metabolic fitness can actively shape epithelial integrity rather than merely responding to barrier failure (102). Conversely, epithelial-intrinsic metabolic pathways also regulate immune accessibility at mucosal surfaces. The HIF target ATG9A is essential for tight junction biogenesis and epithelial barrier maintenance, coordinating autophagy related membrane trafficking with junctional organization (103). By controlling permeability and the translocation of microbial metabolites and antigens, epithelial metabolic programs directly influence downstream inflammatory signaling and immune activation (104). Together, these findings establish epithelial regulation as a bidirectional immunometabolic process integrating immune cell metabolism with epithelial stress-adaptation pathways.

Systemic metabolic signals also contribute to epithelial gatekeeping. Insulin receptor signaling in IECs has been shown to regulate tight junction organization and barrier integrity, linking systemic metabolic status to epithelial permeability (105). Epithelial gatekeeping is further coupled to mitochondrial competence. Perturbations in tRNA modification disrupt mitochondrial function in intestinal epithelial cells, leading to impaired barrier maintenance and concomitant alterations in microbial community structure (106). Defective mitochondrial metabolism alters epithelial oxygen consumption, redox balance, and antimicrobial programs, thereby reshaping the luminal niche in ways that promote dysbiosis and microbiota dependent inflammatory injury (104, 107). These findings suggest that epithelial metabolic stress can act upstream of microbial community disruption, positioning mitochondrial fitness as a determinant of epithelial integrity and microbiome composition. Consistent with this concept, epithelial metabolic dysfunction has been shown to operate at the interface of microbial dysbiosis and tissue injury, where impaired mitochondrial function amplifies inflammatory signaling and epithelial damage, thereby reinforcing maladaptive immune responses (108).

Beyond cell-intrinsic metabolic stress pathways, higher order physiological rhythms impose additional constraints on barrier-microbiota interactions. Circadian alignment coordinates epithelial metabolic processes such as nutrient uptake, mucus secretion, and redox balance with microbial activity. Experimental disruption of light-dark cycles induces visceral hypersensitivity, compromises barrier function, and alters microbial diversity and composition, highlighting circadian regulation as a systems-level modulator of epithelial permeability and immune exposure (109). These observations emphasize that temporal organization of metabolism is integral to maintaining barrier controlled immunometabolic balance.

Specific microbial species can also subvert epithelial gatekeeping by activating regulated cell death programs that secondarily dismantle tight junction control. Periodontitis associated *Fusobacterium nucleatum* has been shown to exacerbate ulcerative colitis by inducing ferroptosis in intestinal epithelial cells, accompanied by loss of junctional proteins such as ZO-1 and claudins and increased intestinal permeability (110). Thus, microbial virulence does not merely provide inflammatory ligands to immune cells but directly destabilizes the epithelial gate that restricts immune access to luminal signals.

Barrier-microbiota-immune interactions are additionally shaped by early life metabolic environments. Maternal metabolic perturbations, including high-fat diet intake, alter fetal intestinal barrier protein expression, induce hypoxic stress, and modulate immune markers, demonstrating that maternal metabolism directly influences epithelial development and immune programming (111). In parallel, maternal microbiota and associated microbial metabolites contribute to fetal intestinal development and immune function (112). Early nutritional environments further regulate epithelial integrity through diet-microbiota interactions, as dietary composition alters microbial communities, epithelial proliferation, and mucosal integrity (113). These findings are supported by broader evidence demonstrating that diet-driven modulation of the microbiota in early life influences intestinal immune regulation (114). Together, these observations indicate that early metabolic environments establish barrier regulated metabolite gradients that imprint immune trajectories beyond the neonatal period. Collectively, these findings establish epithelial barriers as metabolically regulated gatekeepers that integrate microbial, metabolic, and environmental cues to control immune exposure at mucosal surfaces. Intact epithelial regulation supports immune tolerance and tissue integrity, whereas disruption of epithelial metabolic control promotes maladaptive immune activation and chronic inflammation. The role of epithelial metabolic state in regulating tight junction integrity, microbial metabolite access, and downstream immune activation is illustrated in **Figure 3**.

**Figure 3:**
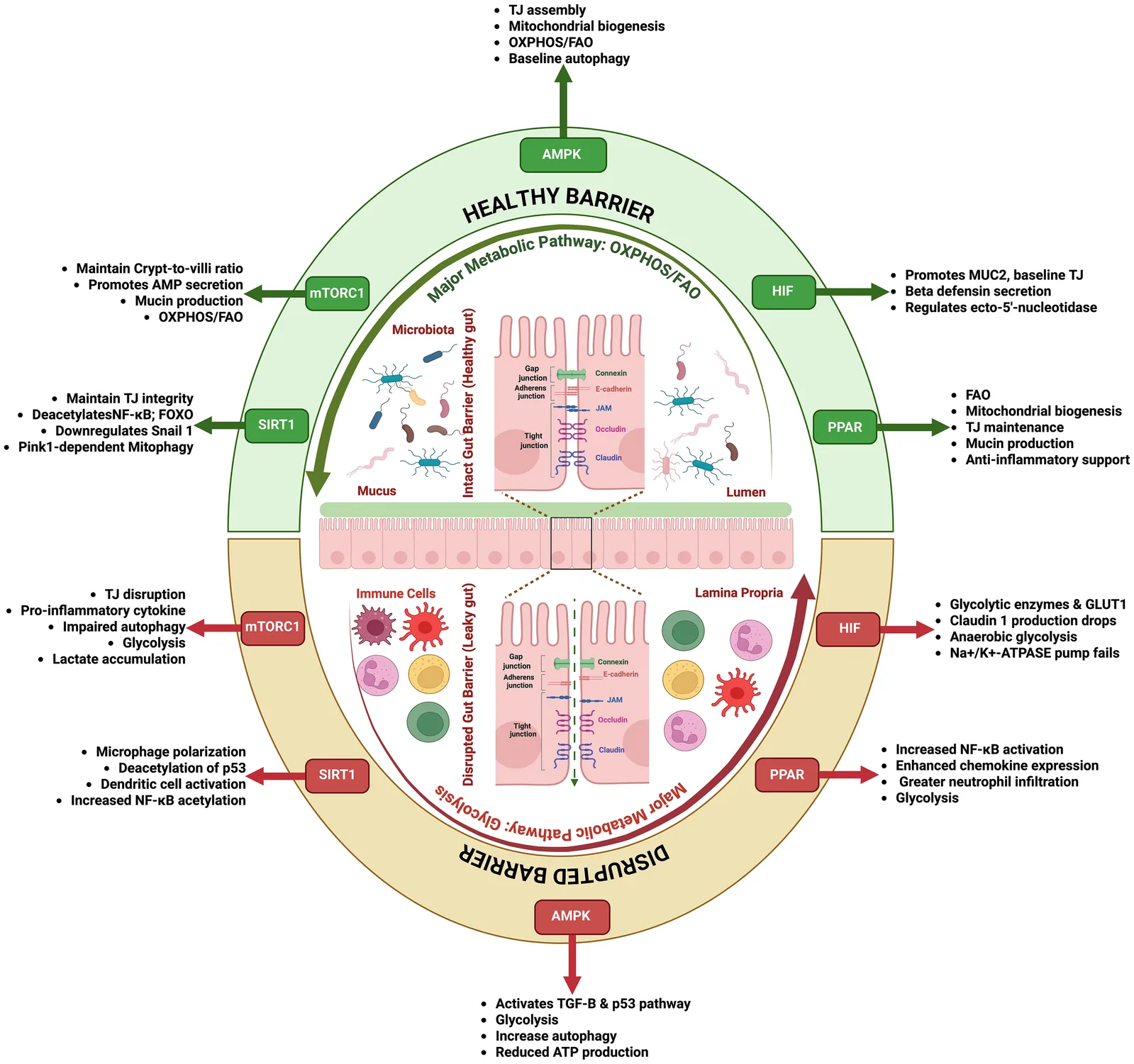
Metabolic control of epithelial barrier integrity. The integrity of the epithelial barrier is regulated by metabolic programs that combine signals derived from microbes with the metabolic sensing of the host. Under normal conditions, oxidative metabolism and the activation of AMPK and SIRT1 promote tight junction assembly and epithelial repair, thereby limiting immune exposure to luminal contents. Metabolites derived from microbiota, such as short-chain fatty acids, bile acids, and tryptophan metabolites, enhance barrier protective pathways. Glycolytic reprogramming, along with dysregulated mTOR or HIF-1α signaling, disrupts tight junction organization, leading to enhanced epithelial permeability and microbial translocation into the lamina propria. This metabolic shift enhances immune activation and amplifies inflammation, establishing epithelial metabolism as a key determinant of immune responses. Created in BioRender. Kumar, V. (2026) https://BioRender.com/noaodx5.

## 5 Barrier regulated immunometabolic dysfunction in disease

Disease associated immunometabolic rewiring frequently emerges from disruption of epithelial metabolic balance and altered barrier-regulated exposure to microbial metabolites. Across chronic inflammatory, autoimmune, metabolic, and neoplastic conditions, conserved metabolic checkpoints including AMPK, mTOR, HIF-1α, PPARs, and SIRT1 integrate microbial, nutritional, and stress derived signals to regulate epithelial permeability, immune activation, and tissue inflammation (115, 116). In this framework, barrier dysfunction acts as a central amplifier of maladaptive immune signaling by increasing permeability and promoting translocation of microbial metabolites, LPS, and inflammatory ligands into tissue and systemic immune compartments. These alterations reinforce chronic inflammation and disrupt tissue integrity across diseases. Short-chain fatty acids derived from intestinal microbiota additionally regulate IL-22 production and mucosal immune responses, further linking microbial metabolite signaling to barrier maintenance and immune regulation (117). Acute tissue injury further illustrates this principle, as CD4^+^ T lymphocytes exhibit biphasic activation and resolution kinetics following myocardial infarction, highlighting temporally ordered immune-state transitions driven by systemic inflammatory cues rather than sustained antigenic stimulation (118).

In obesity associated metaflammation, nutrient overload and dysbiosis synergistically drive persistent inflammatory activation. Under physiological conditions, lean adipose tissue is maintained through AMPK, PPAR, and SIRT1 mediated OXPHOS and fatty acid oxidation in M2 macrophages, Tregs, and eosinophils. Obesity triggers mTOR-driven glycolysis in M1 macrophages and effector T cells, while local hypoxia stabilizes HIF‑1α, reinforcing glycolytic metabolism and pro-inflammatory polarization (115, 119–121). Elevated free fatty acids, adipocyte stress signals, and microbiome derived LPS activate TLR4, NF‑κB, and MAPK pathways, amplifying TNF‑α, IL‑6, and MCP-1 production, and engaging NLRP3 inflammasome signaling (122, 123). Dysbiosis associated depletion of SCFAs, diminishing AMPK and SIRT1 activity, impairing eosinophil mediated tissue remodeling, and promoting barrier dysfunction and metabolic endotoxemia (124, 125). Increased permeability enhances translocation of microbial products such as LPS into systemic circulation, thereby sustaining macrophage activation, inflammatory cytokine production, and chronic low-grade inflammation.

Similar barrier-associated immunometabolic disruptions are observed in autoimmune disease. Tregs depend on AMPK, SIRT1, and PPAR mediated oxidative metabolism to maintain immune tolerance, whereas pathogenic Th17 cells preferentially engage mTOR and HIF-1α-driven glycolysis. Commensal microbiota derived butyrate further supports colonic Treg differentiation and immune tolerance, linking SCFA dependent metabolic signaling to barrier regulated immune balance (35). Dysbiosis associated disruption of epithelial integrity increases translocation of microbial metabolites and inflammatory ligands, thereby amplifying Th17/Treg imbalance and chronic inflammation (126–129). In parallel, leptin and cholesterol intermediates activate mTOR, suppress AMPK, and impair mitochondrial function, reinforcing persistent inflammation and defective immune resolution. Inflammatory bowel disease (IBD) provides a particularly compelling example of barrier-regulated immunometabolic dysfunction, as epithelial metabolic abnormalities directly contribute to chronic intestinal inflammation. In ulcerative colitis, colonocytes exhibit impaired utilization of microbiota derived butyrate, a major oxidative substrate required for epithelial energy production. Early studies demonstrated reduced butyrate oxidation in ulcerative colitis epithelium, supporting the concept that defective epithelial bioenergetics contributes to barrier dysfunction and increased immune exposure to luminal contents (130, 131). More recent evidence further links disrupted mitochondrial dynamics to impaired butyrate metabolism and defective mucosal repair, highlighting the importance of epithelial metabolic fitness in maintaining barrier integrity (132). In contrast, Crohn’s disease is characterized by prominent defects in epithelial microbial handling pathways, including autophagy and mitochondrial quality control. Crohn’s disease susceptibility gene ATG16L1 regulates Paneth cell function, and loss of ATG16L1 activity results in abnormal granule secretion, altered lipid metabolic programs, and impaired antimicrobial defense (133). Defective epithelial stress-adaptation pathways further promote microbial dysbiosis, inflammatory signaling, and barrier disruption. Together, both diseases illustrate distinct but convergent manifestations of epithelial immunometabolic dysfunction, where impaired epithelial energy metabolism, mitochondrial fitness, and host-microbial interactions disrupt barrier function and amplify mucosal immune activation.

Recent evidence further demonstrates that disruption of epithelial mitochondrial dynamics contributes directly to intestinal inflammatory disease. STARD7 dependent maintenance of epithelial mitochondrial architecture is required for preservation of barrier integrity and protection against experimental colitis (134). Similarly, epithelial OPA1-dependent mitochondrial fusion is required for maintaining epithelial integrity and regulating immune responses, further linking mitochondrial dynamics to inflammatory bowel disease pathogenesis (135). Chronic tissue stress similarly contributes to maladaptive immune remodeling, as dysfunctional cardiac microenvironments in dilated cardiomyopathy correlate with altered T-cell phenotypes and diminished effector function under sustained metabolic and inflammatory stress (136). Persistent inflammatory signaling can additionally impose pathological immune fate decisions, as TNFR1 dependent activation induced cell death selectively eliminates aberrantly activated CD4^+^ T lymphocytes during ischemic heart failure (137). In cancer, these mechanisms are further intensified by nutrient competition, barrier dysfunction, and chronic inflammatory remodeling. Tumor cells consume glucose, amino acids, and lipids, generating nutrient deprived and hypoxic microenvironments that suppress mTOR-dependent glycolysis in CD8^+^ T cells, favor AMPK-mediated stress adaptation, and activate HIF-1α-driven hypoxia programs. Tregs adapt via fatty acid oxidation and mitochondrial metabolism, maintaining suppressive function, while myeloid-derived suppressor cells utilize lipid accumulation to enforce immune suppression (138, 139). Sustained immunometabolic imbalance in can also redirect immune programs toward pathological tissue remodeling, with chronic immune-stromal crosstalk reinforcing fibrotic and immunosuppressive microenvironments rather than effective immunity (140). Tumor associated epithelial remodeling may additionally promote local accumulation of KLRG1^+^ GATA3^+^ regulatory T-cells, further linking epithelial alterations to tissue-specific immune suppression and impaired antitumor immunity (141). Altered microbial metabolite signaling additionally influences CD8^+^ T-cell fitness and responsiveness to immune checkpoint blockade. Consistent with this concept, tumor-associated barrier dysfunction and altered microbial metabolite availability may reshape inflammatory signaling and immune metabolism, thereby modulating responsiveness to antitumor immunotherapy (142). In this setting, microbiome derived SCFAs and bile acids activate AMPK, enhance mitochondrial fitness, and restrain mTOR hyperactivation, improving effector T-cell function and response to checkpoint blockade (143–145).

Collectively, these disease examples demonstrate how disruption of epithelial metabolic gatekeeping reshapes immune cell metabolism and inflammatory signaling across diverse pathological settings. Across obesity, autoimmunity, and cancer, conserved metabolic pathways including AMPK, mTOR, HIF-1α, PPARs, and SIRT1 integrate microbial, nutritional, and stress associated signals to regulate epithelial integrity and downstream immune programming. SCFAs, bile acids, and tryptophan metabolites provide critical extrinsic modulation of these pathways, while disruption of barrier regulated metabolite sensing amplifies chronic inflammation, immune dysregulation, and tissue injury. These findings position epithelial metabolic gatekeeping as a central determinant of immune regulation, disease progression, and therapeutic responsiveness.

## 6 Conclusion

Immunometabolism provides a systems-level framework linking environmental cues, cellular energy states, and immune fate decisions. Immune responses are shaped not only by antigen recognition and cytokine signaling but also by regulated exposure to microbiome derived metabolites and tissue derived metabolic cues. Importantly, the immunological effects of these metabolites are not fixed but depend on metabolite availability, epithelial barrier integrity, tissue context, and the metabolic plasticity of responding immune cells.

A central concept emerging from recent work is that epithelial barriers function as active metabolic gatekeepers rather than passive physical boundaries. Through tight junction regulation, transporter activity, stress-adaptation pathways, circadian coordination, and mitochondrial function, epithelial cells serve as critical gatekeepers that regulate the exposure of immune compartments to microbial metabolites and inflammatory ligands. This barrier-controlled metabolite exposure determines whether microbial derived signals promote immune tolerance, tissue repair, and regulatory immune programs or instead drive chronic inflammatory activation and immune dysregulation. Increasing evidence therefore positions epithelial metabolic dysfunction as an upstream determinant of immune pathology rather than merely a downstream consequence of inflammation.

Microbiome derived metabolites including SCFAs, bile acids, tryptophan catabolites, and polyamines converge on conserved metabolic checkpoints such as AMPK, mTOR, HIF-1α, PPARs, and SIRT1 to shape immune activation, mitochondrial fitness, barrier integrity, and tissue-specific immune responses. Importantly, these pathways operate within adaptive and interconnected metabolic networks rather than rigid glycolytic or oxidative states, allowing immune and epithelial cells to dynamically respond to environmental stress, nutrient availability, and inflammatory cues across tissues and disease contexts. Across obesity, autoimmunity, cancer, and chronic inflammatory disorders, disruption of epithelial metabolic balance increases permeability and microbial translocation, thereby reshaping immune checkpoint engagement, inflammatory signaling, and disease progression.

Future challenges include mapping the spatial and temporal organization of immunometabolic networks using single-cell profiling, spatial metabolomics, and barrier-state assessment. Circadian rhythms and developmental windows likely influence immune metabolic programming but remain underexplored. An additional priority will be distinguishing causal from adaptive microbiome-metabolite-immune interactions in chronic inflammatory disease. Barrier associated B-cell responses represent another important frontier, as epithelial signaling and microbial metabolites likely shape mucosal antibody responses and immune memory in ways that remain poorly characterized.

Advancing the field will require moving beyond static and compartmentalized models of immunometabolism toward integrated frameworks that incorporate microbial ecology, epithelial gatekeeping, mitochondrial adaptation, metabolite accessibility, and systemic immune regulation. Such approaches may offer new therapeutic opportunities by restoring epithelial metabolic function, modulating microbiome-derived metabolites, and targeting conserved metabolic checkpoints. Collectively, these findings position epithelial metabolic gatekeeping as a central organizing principle linking microbial metabolism to immune balance, chronic inflammation, and therapeutic responsiveness across disease states.
